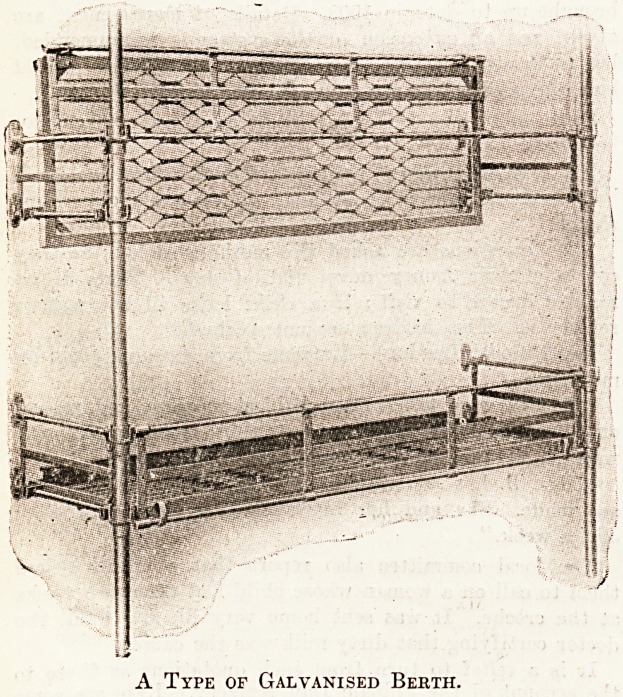# Hospital Accommodation on Merchant Ships

**Published:** 1912-04-20

**Authors:** 


### HOSPITAL ACCOMMODATION ON MERCHANT SHIPS.

The Marine Department of the Board of Trade has issued the following official notice to shipowners: "The attention of shipowners is called to the following recommendation made by a Committee appointed by the Board of Trade to revise the medical scales for merchant vessels."

A Type of Galvanised Berth.

1. Our attention has been called to the absence on board cargo-vessels of any separate accommodation for members of the crew who may fall ill or meet with accidents.

2. We are of opinion that it would be very desirable that some suitable provision should be made on board all ocean-going cargo-vessels for the separate and reserved accommodation of sick persons, and trust the Board will see fit to use its influence with shipowners in this direction.

The Board of Trade strongly supports this recommendation, and as it is framed in the interests of owners as well as employees, and, further, as the majority of owners on inquiry are found to concur, they feel confident that it will commend itself for adoption.

The Board further wish it to be generally known that where such accommodation is provided to the satisfaction of its surveyors as regards floor area, cubic capacity, construction, ventilation, etc., it is entitled to have such accommodation certified as "crew's hospital," and included with the deductions made on account of crew space from the gross tonnage of the vessel. Our illustration shows a type of galvanised berth suitable for this purpose which is arranged to fold when not in use. The front lee-rail, which is attached to the bottom frame, may be disengaged from the cups on the vertical supports and turned down so that the surgeon can conveniently attend the patient. We understand that this berth is made by Messrs. Hoskins and Son, of Birmingham.

## Figures and Tables

**Figure f1:**